# GCN5 HAT inhibition reduces human Burkitt lymphoma cell survival through reduction of MYC target gene expression and impeding BCR signaling pathways

**DOI:** 10.18632/oncotarget.27226

**Published:** 2019-10-08

**Authors:** Aimee T. Farria, Lisa Maria Mustachio, Zeynep H. Coban Akdemir, Sharon Y.R. Dent

**Affiliations:** ^1^ Department of Epigenetics and Molecular Carcinogenesis, The University of Texas MD Anderson Cancer Center, Smithville, Texas 78957, USA; ^2^ The Center for Cancer Epigenetics, The University of Texas MD Anderson Cancer Center, Smithville, Texas 78957, USA; ^3^ Program in Genetics and Epigenetics, The University of Texas MD Anderson Cancer Center, UT Health Graduate School of Biomedical Sciences, Houston, Texas 77030, USA; ^4^ Departments of Molecular and Human Genetics, Baylor College of Medicine, Houston, Texas 77030, USA

**Keywords:** GCN5, MYC, lymphoma, BCR

## Abstract

GCN5, the catalytic subunit in the acetyltransferase modules of SAGA and ATAC, functions as a coactivator of gene transcription. The SAGA complex is recruited to chromatin by transcription factors such as MYC and E2F1 to facilitate acetylation of histones, especially H3 at lysine 9 (H3K9). Burkitt lymphoma is an aggressive subtype of Non-Hodgkin lymphoma driven by the overexpression of MYC. Comparison of GCN5 expression in normal human B cells versus human Burkitt Lymphoma cell lines indicates overexpression of GCN5 in lymphoma. Treatment of Burkitt lymphoma cell lines with a specific inhibitor indicates that decreased GCN5 HAT activity reduces viability and proliferation of these cells. Inhibition of GCN5 HAT activity also induces apoptosis in lymphoma cells. Expression of MYC target genes as well as genes associated with B cell receptor signaling are significantly downregulated upon inhibition of GCN5 enzymatic activity. This downregulation leads to diminished PI3K signaling, a critical pathway in lymphomagenesis. Our data indicate that inhibition of GCN5 HAT activity reduces the tumorigenic properties of human Burkitt lymphoma cells by attenuating BCR signaling and that GCN5 may be a viable target for lymphoma drug therapy.

## INTRODUCTION

Burkitt lymphoma (BL) is a highly aggressive subtype of Non-Hodgkin lymphoma that develops from mature B cells in the germinal center of the spleen. It is promoted by the overexpression of c-MYC caused by the translocation of the *c-MYC* proto-oncogene to the *IgH* locus. Burkitt lymphoma survival is dependent on “tonic” BCR signaling [1, 2]. Tonic signaling provides a basal level of signaling without stimulation from a ligand and is essential for not only BL cell survival but is also important for normal B cell function. Tonic signaling activates the PI3K pathway in BL cells, and the consequent signaling cascade promotes proliferation [1]. Treatment for BL in the developed world entails high doses of chemotherapy and may include targeted drug therapies including anti-CD20 drugs. This treatment is highly successful in children but can be dangerous for older people. Chemotherapy also has limited efficacy in the developing world, which sees increased levels of the endemic form of BL due to the high levels of malaria and Epstein Barr Virus (EBV) infection. For these reasons, more accessible drug therapies are required to improve survival rates.

GCN5 (KAT2A), and its paralog PCAF (KAT2B), are lysine acetyltransferases conserved from yeast to mammalian cells that primarily function as cofactors in transcriptional regulation [3–5]. The role of GCN5 in the function of the Spt-Ada-Gcn5 acetyltransferase (SAGA) complex is very well studied, but GCN5 also resides in the less well-characterized Ada2A-containing (ATAC) complex [6–8]. SAGA and ATAC are recruited to chromatin by transcription factors such as E2F1 and c-MYC [9–12]. There, GCN5 acetylates histones allowing DNA accessibility for the transcriptional machinery.

MYC is a known proto-oncoprotein that is overexpressed in most cancers. Interestingly, Gcn5 has been linked to Myc functions in mouse embryonic stem cells, during somatic cell reprogramming and during mouse neural development [13–15]. GCN5 has been also been implicated in progression of many different cancers, including non-small cell lung cancer, colon cancer and glioma [16–18]. In addition, *GCN5* was identified in a CRISPR screen as one of several genes necessary for the survival of AML cells [19]. Interestingly, GCN5 has also been linked to PI3K signaling [20–22], which works synergistically with MYC in Burkitt lymphoma.

These previous studies led us to hypothesize that GCN5 may play a role in MYC driven cancers. In this study, we sought to ascertain if GCN5 activity contributes to the progression of Burkitt lymphoma. We find that inhibition of GCN5 HAT activity reduces the viability and proliferation of Burkitt lymphoma cells. Moreover, GCN5 inhibition induces apoptosis of the BL cells. We observe that GCN5 HAT inhibition disrupts BCR signaling, possibly by down regulating the expression of Spleen Tyrosine Kinase (SYK), thus down regulating the phosphorylation of AKT and its targets. Expression of several other MYC transcriptional target genes are down regulated upon GCN5 inhibition as well. These findings indicate that GCN5 may provide a viable therapeutic target in Burkitt lymphoma through regulation of MYC and the PI3K pathway.

## RESULTS

### GCN5 is overexpressed in human Burkitt lymphoma

**Figure 1:**
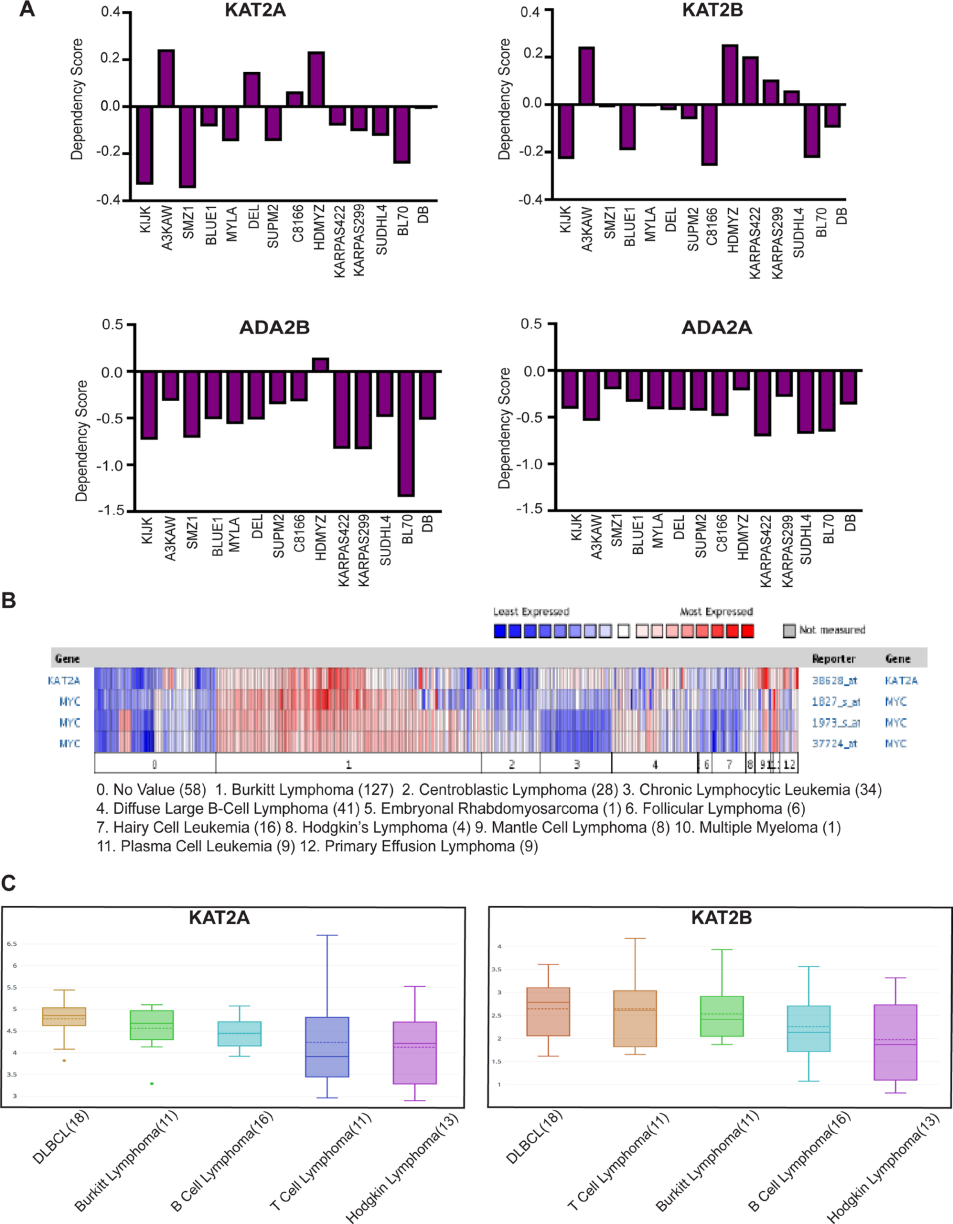
GCN5 and SAGA is implicated in human Burkitt Lymphoma. **(A)** CERES Dependency Scores of CRISPR knockout *KAT2A, KAT2B, ADA2B* and *ADA2A* lymphoma cell lines profiled by DepMap (Broad Institute). **(B)** Expression of GCN5 and MYC was compared using the Oncomine database in the cancers shown above. Numbers in parentheses indicate the numbers of cases reported. **(C)** Graph of mRNA level of KAT2A and KAT2B in lymphoma cell lines reported by CCLE database.

We began our studies by taking advantage of a publicly available database, the Cancer Dependency Map from the Broad Institute (https://depmap.org/portal/), to determine whether *GCN5*, *PCAF*, or other components of SAGA and ATAC are important for lymphoma cell survival. This database identifies genes required for cancer cell survival or growth using CRISPR or shRNA mediated knockout/knockdown of individual genes. Dependency scores between -0.4 and -1 are categorized as significant. Examination of CRISPR screen data revealed some dependence of leukemia and lymphoma cell lines on either or both *KAT2A* (*GCN5)* and *KAT2B* (*PCAF)* (Figure 1A). The overall weakness in dependency on either individual factor might reflect redundancy in functions of these HATs. Therefore, we also examined dependencies of lymphoma cells on *ADA2B* and *ADA2A,* which encode important components of the HAT modules of the SAGA complex (ADA2B) and the ATAC (ADA2A) complex. Loss of ADA2B or ADA2A abrogates HAT activity of both GCN5 and PCAF containing versions of SAGA and ATAC. Consequently, stronger dependency scores were observed for *ADA2B* and *ADA2A* in leukemia and lymphoma cells than for either *GCN5* or *PCAF* (Figure 1A). In general, these cell lines showed greater dependency on *ADA2B* than on *ADA2A*, indicating SAGA may be especially important for survival of leukemia and lymphoma cells.

Next, we explored the Oncomine database to determine whether GCN5 expression is altered in hematopoietic cancers. We found that GCN5 (KAT2A) mRNA is overexpressed in certain lymphomas, and especially in Burkitt lymphomas, which have high expression of MYC (Figure 1B). We also examined expression of GCN5 (KAT2A) and PCAF (KAT2B) in lymphoma cell lines in the Cancer Cell Line Encyclopedia (CCLE) database and again observed that GCN5 (KAT2A) mRNA levels are elevated in many different cancer cell lines including human Burkitt lymphoma (BL) cells (Figure 1C). PCAF levels are also elevated, although to a lesser degree (Figure 1C). These results further suggest that these HATs may be important for the survival of Burkitt lymphoma cells.

### Inhibition of GCN5 HAT activity reduces viability of Burkitt lymphoma cells

**Figure 2:**
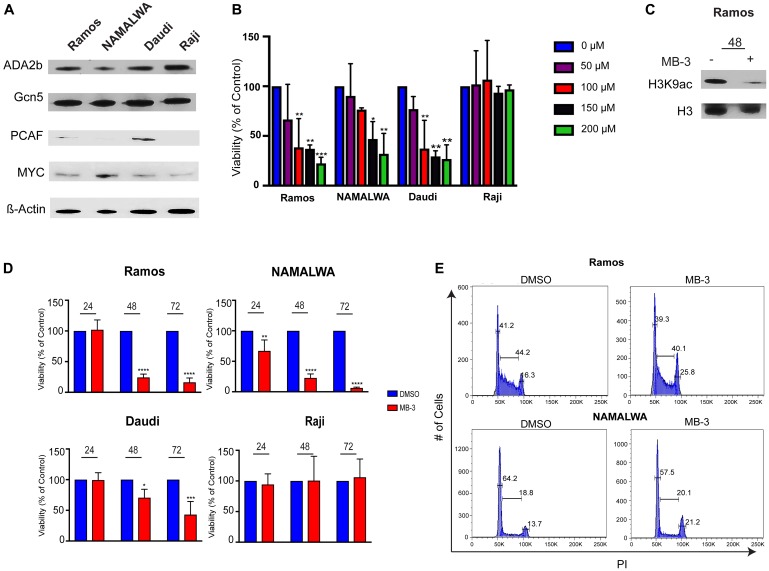
GCN5 HAT Inhibition Reduces Viability of human Burkitt lymphoma cell lines. (**A**) Protein levels of SAGA components were detected in 4 Burkitt lymphoma cell lines using immunoblots. (**B**) IC50 of MB-3 in BL cell lines determined by Cell Titer Glo viability assay after 48 hours. The data indicate mean ± SD of three experiments. (**C**) Histones were extracted from Ramos cells and H3K9ac and H3 measured by immunoblot. (**D**) Cell viability assessed at 24, 48, and 72 hours by Cell Titer Glo assay. The data indicate mean ± SD of three experiments. ^*^ *p* ≤ 0.05; ^**^ *p* ≤ 0.01; ^***^ *p* ≤ 0.001; ^****^ *p* ≤ 0.0001. Significance determined by two-way ANOVA. (**E**) Cell cycle distributions were assessed by staining cells with propidium iodide and measuring by FACS. Representative histogram of three experiments demonstrates cells in each phase of cell cycle.

To determine the physiological effects of loss of GCN5/PCAF activity, we treated several human BL cell lines with the GCN5/PCAF specific inhibitor Butyrolactone 3 (MB-3), after confirming that GCN5 and PCAF, as well as other SAGA components, are expressed in these BL cell lines (Figure 2A). GCN5 and ADA2B were well expressed in all four cell lines examined, whereas PCAF expression was more variable. MB-3 has a reported IC50 of 100 μM for binding to the GCN5 HAT domain [23], so we determined the sensitivity of the different cell lines to inhibitor treatment using a range of drug between 50-200 μM. We found that Ramos, NAMALWA, and Daudi cells were sensitive to the inhibitor in a concentration dependent manner. The Raji cell line was insensitive to the inhibitor at all doses tested (Figure 2B). Immunoblots of H3K9 acetylation levels in Ramos cells verified inhibition of HAT activity after 48 hours of exposure to MB-3 (Figure 2C).

We next measured the viability of BL cells treated with 100 μM MB-3 over time. HAT inhibition led to loss of viability of Daudi, Ramos and NAMALWA cell lines at 48 and 72 hours, whereas Raji cell viability was unaffected (Figure 2D, Supplementary Figure 1A). Cell cycle analysis of the Ramos and NAMALWA cell lines revealed a reduction of cells in the G1 phase, with an increase of cells in the G2, consistent with delayed progression through G2/M. G2/M delay was noted previously upon loss of *GCN5* in both yeast and mammalian cells [24, 25]. Expression of CDC25B (Supplementary Figure 1B), which is both a MYC target gene in Burkitt lymphoma and necessary for G2/M transition, was decreased upon MB-3 exposure [26]. Taken together, these data indicate that GCN5/PCAF inhibition reduces proliferation and causes cell cycle delay in BL cells.

### Inhibition of GCN5 HAT activity induces apoptosis in Burkitt lymphoma cells

**Figure 3:**
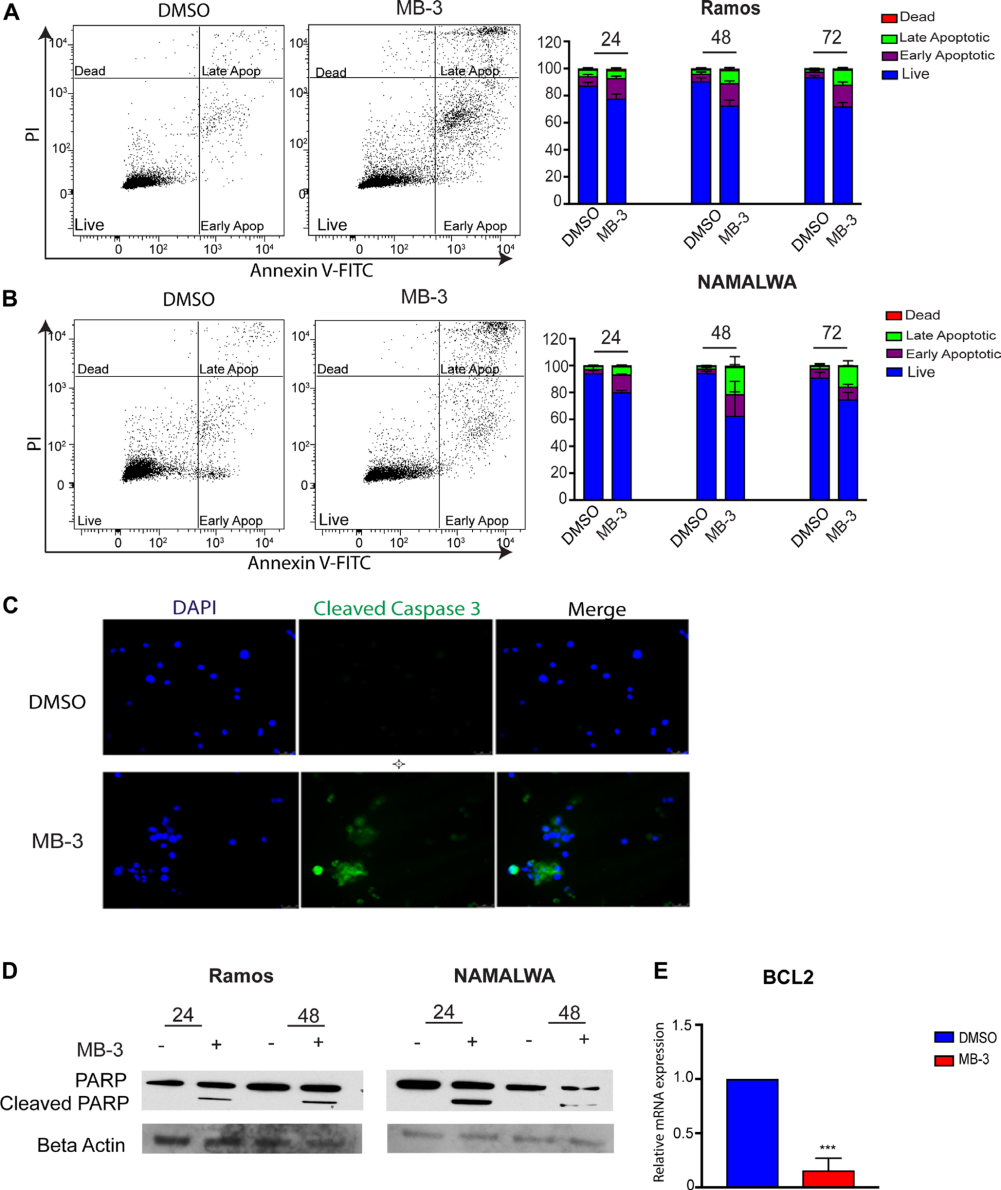
GCN5 inhibition induces apoptosis in human Burkitt lymphoma cell lines. (**A**) Apoptosis of Ramos and (**B**) NAMALWA cell lines was assessed upon treatment of DMSO and 100 μM MB-3 at 24, 48, and 72 hours by Annexin V/PI FACS assay. The bar graph indicates mean ± SD of three experiments. ^*^ *p* ≤ 0.05; ^**^ *p* ≤ 0.01; ^***^ *p* ≤ 0.001; ^****^ *p* ≤ 0.0001. Significance determined by two-way ANOVA. (**C**) Representative fluorescent images of Ramos cells were stained with cleaved caspase 3 and DAPI. (**D**) Cleaved PARP was detected by immunoblot in Ramos and NAMALWA cell lines. (**E**) mRNA levels of BCL2 in Ramos cells was measured by qRT-PCR.

Previous reports indicated that loss of GCN5/PCAF induced apoptosis in leukemia cells [27] and lung cancer stem-like cells [16]. Our proliferation assays indicated that inhibition of GCN5/PCAF might be causing cell death in BL cells as well as delays in cell cycle progression (Supplementary Figure 1A). Cell sorting analyses further indicated that inhibition of GCN5/PCAF activity resulted in increased apoptosis, with a significant number of cells becoming necrotic (late apoptotic) by 72 hours (Figure 3A–3B). Immunofluorescence staining for cleaved caspase 3 also indicated an increase in apoptotic cells upon MB-3 treatment (Figure 3C), and immunoblots revealed elevated cleavage of PARP (Figure 3D). Interestingly, MYC driven cancer cells can avoid MYC- induced apoptosis by loss of p53 expression or function or by gain of expression of the anti-apoptotic factor BCL-2 [28–30]. Many Burkitt lymphoma cell lines have mutant *p53* [31, 32], so we investigated BCL2 transcript levels and found that MB-3 inhibition downregulated BCL2 expression in Ramos cells (Figure 3E). These findings indicate that GCN5/PCAF HAT inhibition induces cell death in BL cells.

### Loss of GCN5 activity alters expression of PI3K effectors

**Figure 4:**
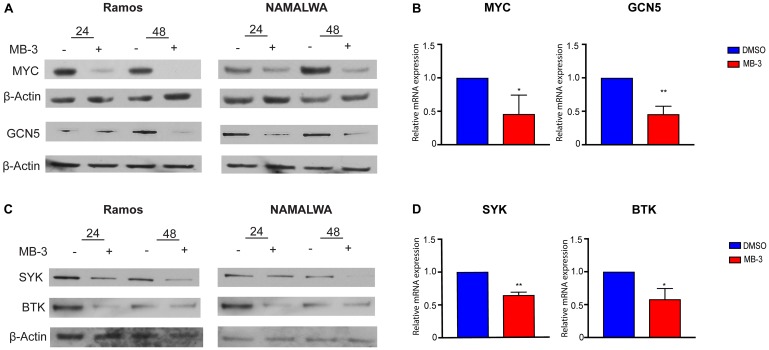
MYC target genes downregulated upon inhibition of GCN5/PCAF HAT activity. (**A**) Representative immunoblots to measure MYC and GCN5 protein levels following 24 and 48 hours of treatment. (**B**) MYC and GCN5 mRNA levels of Ramos cells were measured by qRT-PCR. (**C**) Representative immunoblots measured SYK and BTK protein levels following 24 and 48 hours of treatment. (**D**) SYK and BTK mRNA levels of Ramos cells were measured by qRT-PCR. Error bars show mean ± SD of three replicates. All *p*-values determined by unpaired *t*-test. ^*^ *p* ≤ 0.05; ^**^ *p* ≤ 0.01; ^***^ *p* ≤ 0.001; ^****^ *p* ≤ 0.0001.

GCN5 and PCAF are well known for their roles as transcriptional co-activators. A previous study identified GCN5 as necessary for the transcription of Spleen Tyrosine Kinase (SYK) as well as a downstream effector of SYK, Bruton’s Tyrosine Kinase (BTK), in chicken B cells [20]. SYK is an integral component of the B cell signaling pathway whose phosphorylation activates PI3K signaling. SYK is also a transcriptional target gene for c-MYC [33–35], and it may regulate the expression of MYC [35]. We found that MB-3 mediated inhibition of GCN5/PCAF activity lead to a reduction of SYK, BTK, and MYC expression, both at the RNA and protein levels, in BL cells (Figure 4A–4D). GCN5, which is itself a transcriptional target of MYC, was also downregulated. Our results indicate that MB-3 treatment interrupts a gene expression program controlled by a regulatory loop of interconnected MYC-SYK-GCN5 functions.

### GCN5 HAT inhibition attenuates tonic and active BCR signaling

MYC overexpression and PI3K signaling have been linked in Burkitt Lymphoma and are both necessary for the survival and fitness of BL cells [1]. Previous studies have linked GCN5 both to MYC target gene transcription and to PI3K signaling [18, 20, 21]. GCN5 has also been linked to BCR signaling [36]. Therefore, we sought to determine if MB-3 inhibition of GCN5 activity disrupts these crucial pathways in Burkitt lymphoma.

**Figure 5:**
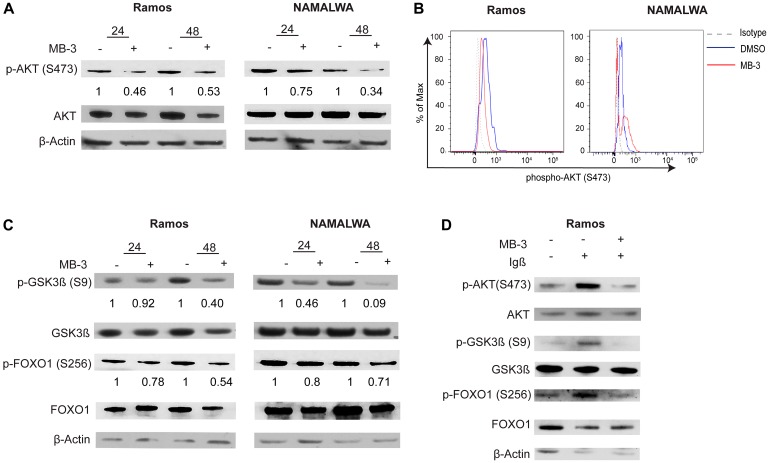
GCN5 inhibition attenuates PI3K signaling in human Burkitt lymphoma cell lines. (**A**) Representative immunoblots are shown to assess phospho-AKT (S473) levels after treatment for 24 and 48 hours. Levels were compared to total AKT. (**B**) Phospho-flow cytometry was used to measure levels of phospho-AKT (S473) in Ramos and NAMALWA cells after treatment for 48 hours. Histogram representative of three experiments. (**C**) Representative immunoblots measured phosphorylation of AKT substrates GSK3β(S9) and FOXO1(S256). Levels were measured against total protein levels. (**D**) Representative immunoblots measured phospho-AKT, phospho-GSK3β(S9) and phospho-FOXO1(S256) from BCR stimulated Ramos cells. Levels were measure against total protein levels.

We observed a significant decrease in AKT phosphorylation in unstimulated BL cells, indicating that MB-3 treatment diminishes tonic BCR signaling (Figure 5A–5B). Interestingly, we also observed a decrease in the amount of total AKT. AKT can be cleaved by active caspase 3 [37], subsequently the increase in cleaved caspase 3 we observed upon MB-3 treatment (Figure 3C) might contribute to increased AKT degradation. The decrease in AKT levels also leads to a decrease in AKT signaling. Phosphorylation of AKT substrates GSK3β and FOXO1 was also decreased upon MB-3 treatment (Figure 5C), further confirming disruption in PI3K signaling upon inhibition of GCN5/PCAF HAT activity in BL cells.

Next we examined if GCN5 inhibition attenuated active BCR signaling. Igα (CD79a) and Igβ (CD79b) are integral components in BCR signaling [38]. A recent study has also shown that an alternative signaling module with the association of Igβ and CD19 is essential for the fitness of Burkitt lymphoma cells and subsequent PI3K signaling [39]. We used anti-Igβ to stimulate the BCR in RAMOS cells with or without MB-3 treatment. Decreased levels of AKT and phospho-AKT, and decreased phosphorylation of downstream AKT substrates GSK3β, and FOXO1 were observed upon GCN5/PCAF inhibition (Figure 5D).

Together, our results indicate that GCN5/PCAF inhibition attenuates BCR signaling in lymphoma cells, including tonic signaling upon which BL cells depend.

## DISCUSSION

Our results strongly indicate that GCN5/PCAF promotes survival of Burkitt lymphoma cells through the promotion of B cell receptor signaling. Our study links MYC to BCR signaling in Burkitt lymphoma through up regulation of *SYK* transcription by MYC and GCN5. SYK is an essential component of the BCR signaling pathway that links the BCR to a downstream kinase cascade including phosphorylation of PI3K and AKT. SYK inhibition has been shown to reduce the viability of Non-Hodgkin lymphoma cells by disruption of BCR signaling [2, 40, 41]. We demonstrated that inhibition of GCN5 activity disrupts the BCR signaling cascade by multiple modes. Reduction of SYK transcription and protein levels disrupts the SYK-PI3K-AKT axis [42]. In addition, reduction of AKT levels and phosphorylation of AKT disrupts downstream phosphorylation of AKT substrates, including GSK3β and FOXO1. Phosphorylation of GSK3β by AKT inactivates GSK3β kinase activity, and this inhibition is necessary for the fitness of MYC-driven lymphoma cells [43]. FOXO1 is a tumor suppressor in B cell malignancies [44]. It acts as a transcription factor involved in regulating genes involved in regulating cell cycle and inducing apoptosis among other cellular processes [45–47]. Phosphorylation of FOXO1 by AKT inactivates the protein and sequesters it in the cytosol, which leads to a decrease in target gene expression [48, 49]. A recent study reported that nuclear FOXO1 has an oncogenic function in BL when paired with constitutively active PI3K signaling [50]. By decreasing BCR signaling, the activity of the PI3K signaling pathway is reduced, cancelling out this survival advantage, an effect also seen previously [50]. Interestingly, the Raji cell line was resistant to the MB-3 inhibitor. Previous studies indicate that this cell line is drug insensitive, particularly to inhibitors of the PI3K and BCR pathways [51, 52]. Overall, GCN5 HAT inhibition disrupts BCR signaling and reduces phosphorylation of key effectors, thereby reducing viability and inducing apoptosis in Burkitt lymphoma cells.

No effective means currently exists for directly targeting the oncoprotein MYC in cancer. Alternative approaches seek to circumvent MYC functions by either reducing MYC expression levels or by attacking MYC partners in order to reduce its capacity to induce transcription [53]. GCN5 provides an attractive target for achieving both of these goals. Acetylation of MYC by GCN5, and other HATs, protects MYC from degradation. In addition, active GSK3β phosphorylates c-MYC, marking the protein for degradation [54]. We have shown that MB-3 treated cells have reduced MYC protein levels. GCN5 is also an important co-factor for MYC driven transcription programs. The disruption of the GCN5-MYC transcriptional feedback loop leads to reduced mRNA levels of both GCN5 and MYC. The degradation of the MYC protein, combined with transcriptional downregulation of SYK, GCN5, and MYC, significantly reduces the cancer promoting properties of MYC.

Inhibition of GCN5/PCAF may have multiple effects on Burkitt lymphoma survival or progression. MYC cooperates and enhances PI3K signaling in Burkitt lymphoma through multiple modes [50, 55]. Targeting GCN5 inhibits both the cancer promoting effects of MYC-driven transcription programs and disrupts PI3K signaling. In the future, inhibition of GCN5 as well as SYK with targeted drug therapies may provide a synergistic therapy for Non-Hodgkin lymphoma. Combination of GCN5 inhibition, to limit MYC functions, with bromodomain inhibitors, to limit MYC expression, may also improve efficacy. Such effects are likely limited not to Burkitt lymphoma but are also relevant to other MYC-addicted cancers. Unfortunately, currently there are no clinically active molecules that effectively and selectively target GCN5/PCAF. Our results highlight the need for the development of such drugs.

## MATERIALS AND METHODS

### Bioinformatics analysis

DepMap (Broad Institute, https://depmap.org/portal/) was explored for dependencies (CERES score) of *ADA2A*, *ADA2B*, *KAT2A*, and *KAT2B* in CRISPR lymphoma cell lines from the CRISPR (Avana) Public 19Q2 dataset.

GCN5 and MYC mRNA expression data from leukemia, lymphoma, and multiple myeloma patient samples were analyzed using the Oncomine database (https://www.oncomine.org).

mRNA level of KAT2A and KAT2B in lymphoma cell lines was analyzed using the Cancer Cell Encyclopedia (CCLE) database (Broad Institute, https://portals.broadinstitute.org/ccle).

### Cell culture and reagents

Human Burkitt lymphoma cell lines Ramos, Daudi, NAMALWA, and Raji were purchased from ATCC. All cell lines were validated by the Characterized Cell Line Core Facility at MD Anderson using Short Tandem Repeat DNA profiling (STR fingerprinting). Ramos, Daudi, and Raji cell lines were maintained in RPMI 1640 with 10% FBS and 1% Penicillin-Streptomyocin. NAMALWA cells were maintained in RPMI 1640 with 7.5% FBS, 2 mM L-glutamine, 1.5 g/L sodium bicarbonate, 4.5 g/L glucose, 10mM HEPES, and 1.0 mM sodium pyruvate. DMSO (Sigma) was used as a vehicle. Butyrolactone-3 (MB-3) inhibitor was purchased from Cayman Chemical and dissolved in DMSO.

### Cell viability

Cells were plated in 96-well plates at a concentration of 10,000 cells per well. 24 hours later, cells were treated with vehicle (DMSO) or 100 μM MB-3 inhibitor. Viability was measured at 24, 48, and 72 hours post treatment using Cell Titer Glo^®^ Luminescent Cell Viability Assay (Promega) per manufacturer instructions. Luminescence was measured using a FLUOstar Omega microplate reader (BMG Labtech).

### Flow cytometry

All flow cytometry analyses were performed on a BD LSRFortessa and data were analyzed with FlowJo software.

### Apoptosis analysis

Cells were plated in 48-well plates at 50,000 cells per well. 24 hours later cells were treated with vehicle (DMSO) or MB-3 inhibitor. Cells were collected at 24, 48 and 72 hours and pelleted. Pellets were washed in 1× PBS and resuspended in Annexin V buffer (10 mM HEPES (pH 7.4),150 mM NaCl, and 2.5 mM CaCl_2_ in 1× PBS). Cells were stained with Annexin V-FITC (Biolegend) and propidium iodine (Life Technologies) and allowed to incubate for 15 minutes at room temperature. Cells were then analyzed by flow cytometry.

### Cell cycle analysis

Cells were plated in a 48-well plate at 50,000 cells per well and incubated for 24 hours. 24 hours later treated with vehicle (DMSO) or MB-3 inhibitor. Cells were collected at 24, 48 and 72 hours and pelleted. Pellets were washed in 1× PBS and there resuspended in 100% ethanol and incubated at -20 degrees Celsius overnight. Cells were allowed to incubate on ice for 30 minutes and then centrifuged at 4 degrees Celsius and resuspended in 1× PBS with 20 μg/ml RNase A and again incubated on ice for 30 minutes. Cells were stained with propidium iodine to a final concentration of 20 μg/ml in 1× PBS and allowed to incubate for 30 minutes. Cells were then analyzed by flow cytometry.

### BCR stimulation

Ramos cells were treated with DMSO or 100 μM MB-3 for 48 hours. Cells were washed in 1× PBS and spun down for 5 minutes at 300 at room temperature. Cells were starved in serum free RPMI for 20 minutes at 37 degrees Celcius 5% CO_2._ 10 μg Anti-CD-79b (R&D Systems) was added to cells to stimulate the BCR for 5 minutes at room temperature. Cells were washed in 1× PBS and lysed for protein purification.

### Immunofluorescence

Cells were plated on poly-l-lysine coated slides. Cells were fixed with 2% PFA and washed in 100% methanol and then incubated in 100% methanol for 5 minutes at 20 degree Celsius. An equal amount of 1× PBS was added and removed and then slides were washed three times with 1× PBS for 5 minutes each. Slides were washed for 5 minutes in washing buffer (1× PBS and 1% Gelatin). Slides were blocked in blocking buffer (1× PBS, 0.5% gelatin, and 1% normal goat serum) at 4 degrees for 30 minutes. Blocking buffer was removed and anti-Cleaved Caspase 3 (1:400; Cell Signaling) diluted in wash buffer added and slides incubated at room temperature for 1 hour. Slides were washed in washing buffer 5 times for 5 minutes each. Secondary antibody diluted in washing buffer was added and slides were incubated at room temperature for 1 hour each. Slides were washed with washing buffer 2 times for 5 minutes each. Slides were stained with DAPI (1:2000) in wash buffer for 5 minutes and washed 2 times for 5 minutes each. VECTASHIELD^®^ Antifade was used as mounting media.

### Protein purification

Cells were harvested, pelleted, and washed in 1× PBS. Pellets were resuspended in Buffer C (20 mM Tris-HCl pH 7.9, 20% glycerol, 420 mM NaCl, 1.5 mM MgCl_2_, 0.1% NP-40, 0.2 mM EDTA, 0.5 mM DTT, 0.2 mM PMSF, and Sigma Protease inhibitors), vortexed and rocked at 4 degrees Celsius for 20 minutes. After rocking, an equal amount of Buffer A (10 mM HEPES pH 7.5, 1.5 mM MgCl_2_, 10 mM KCl) was added. Lysate was centrifuged at 4 degrees Celsius for 10 minutes at 10,000 RPM. Supernatant was collected as the whole cell extract and total protein levels measured by Bradford assay.

### Histone extraction

Histones were extracted using the Histone Purification Kit (Active Motif). Purified histones were measured using spectrophotometer (Nanodrop).

### Immunoblotting

20μg of protein was electrophoresed on 4-12% Nu-Page gel (Life Technologies) and transferred to a nitrocellulose membrane. Membranes were blocked in 5% milk in TBST at room temperature for 30 minutes. Membranes were incubated overnight at 4 degrees Celsius with the primary antibodies: anti-GCN5 (1:1000), anti-MYC (1:1000), anti-AKT (1:1000), anti-phospho-AKT (1:1000), anti-SYK (1:1000), anti-BTK (1:1000), anti-FOXO1 (1:1000), anti-phospho-FOXO1 (1:1000), and anti-PARP (1:1000) (Cell Signaling); anti-Ada2b (1:500); anti-Beta Actin (1:5000) (Santa Cruz), anti-USP22 (1:1000) (homemade); H3 acetyl lysine 9 (1:500) (Millipore); H3(1:10,000) (Abcam). Membranes were incubated in secondary-horseradish peroxidase (HRP) conjugated antibodies (GE Healthcare, Cat. # NA934V for Rabbit, NA931V for Mouse) for 1 hour at room temperature. Amersham ECL Prime Western Blotting Detection Reagent (GE Healthcare) was used for chemiluminescent protein detection.

### RNA isolation and quantitative RT-PCR (qRT-PCR)

Cells (2 × 10^6^) were plated at in 60 mm plates and incubated for 24 hrs. Cells were treated with either vehicle or MB-3 inhibitor. After 24 hours cells were harvested and washed in 1× PBS. Total RNA was isolated using the RNeasy® Purification Kit (Qiagen). Power SYBR^®^ RNA-to-Ct kit (Thermo Fisher) was used to measure mRNA levels and reactions were run in an Applied Biosciences 7500 Fast Block Real Time PCR System. 20 μg of RNA was used in each reaction. Primers sequences are listed in Supplementary Table 1. All experiments were performed three times. ΔΔCT was calculated for all samples and all DMSO controls were set to 1.

### Statistical analysis

All data were represented as mean ± standard deviation. The statistical analyses for qRT-PCR were performed in Microsoft Excel. All other statistical analyses were performed in Prism 7 (GraphPad Software 7.0) using Students *t*-test or analysis of variance. Significant *P*-value was < 0.05.

## SUPPLEMENTARY MATERIALS


