# Supplementary material for: GCN5 HAT inhibition reduces human Burkitt lymphoma cell survival through reduction of MYC target gene expression and impeding BCR signaling pathways
Source: Oncotarget. 2019 Oct 8;10(56):5847–58. doi: 10.18632/oncotarget.27226 (PMC6791378; doi:10.18632/oncotarget.27226)
Supplement: Supplementary file 1 [file oncotarget-10-5847-s001.pdf]

GCN5 HAT inhibition reduces human burkitt lymphoma cell survival through reduction of myc target gene expression and impeding BCR signaling pathways

SUPPLEMENTARY MATERIALS

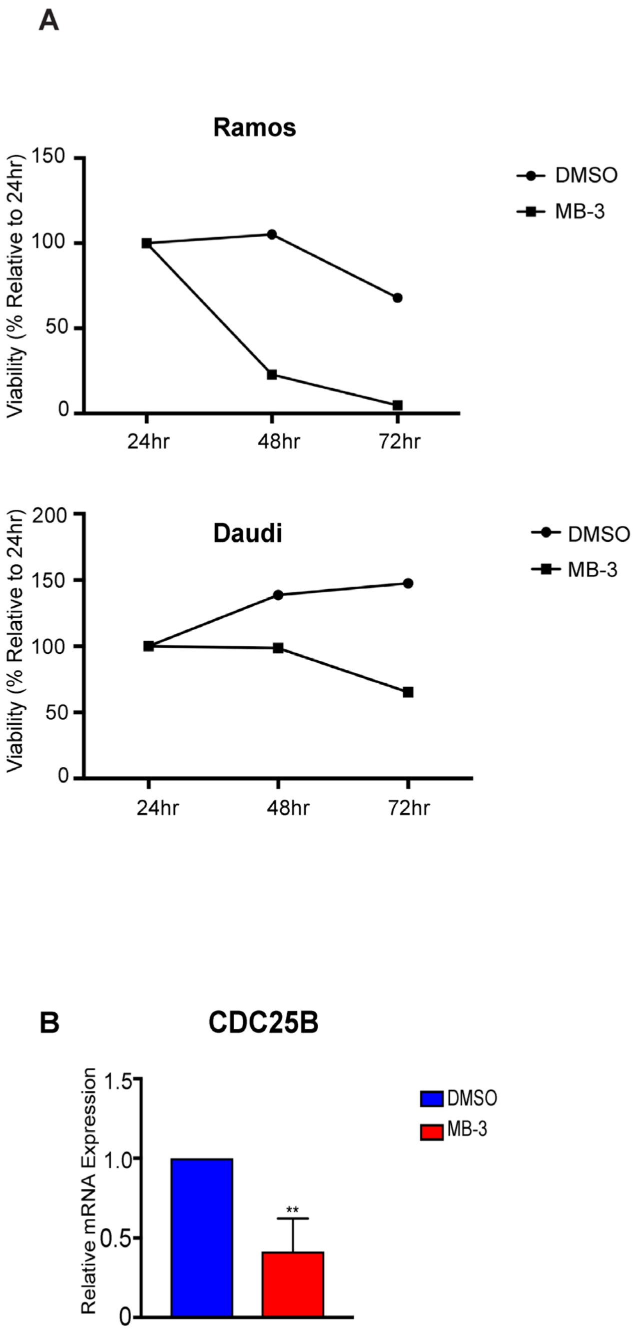

Supplementary Figure 1: GCN5 HAT Inhibition Reduces Proliferation of human Burkitt lymphoma cell lines. (**A**) Cell viability assessed at 24, 48, and 72 hours by Cell Titer Glo assay. The data is representative of three experiments. (**B**) mRNA levels of CDC25B in Ramos cells was measured by qRT-PCR. Error bars show mean ± SD of three replicates. All p-values were determined by unpaired t-tests.

Supplementary Table 1: Primers for qRT-PCR

Gene name	Primer sequence
GCN5 FWD	5′-GTGCTGTCACCTCGAATGAG-3′
GCN5 REV	5′-CGGCGTAGGTGAGGAAGTAG-3′
MYC FWD	5′-TTTCGGGTAGTGGAAAACCA-3′
MYC REV	5′-CACCGAGTCGTAGTCGAGGT-3′
SYK FWD	5′- TTTCGGACTTTCCAAAGCACTGCG-3′
SYK REV	5′- ACTCCAAAGCTCCAGACATCGCTT-3′
BTK FWD	5′- TCTGAAGCGATCCCAACAGAA-3′
BTK REV	5′- TGCACGGTCAAGAGAAACAGG-3′
CDC25B Fwd	5′-GCTCTGGGGAAGACAAGGAGAA-3′
CDC25B Rev	5′-TGGCACTTGCTGTACATGACGA-3′
BCL-2 Fwd	5′-CCTTTGGAATGGAAGCTTAG-3′
BCL-2 Rev	5′-GAGGGAATGTTTTCTCCTTG-3′
Beta Actin FWD	5′-GCCAACCGCGAGAAGATGACC-3′
Beta Actin REV	5′-CTCCTTAATGTCACGCACGATTTC-3′
